# Leveraging industry 4.0 technologies for healthcare innovation and efficiency

**DOI:** 10.1371/journal.pone.0336222

**Published:** 2025-11-05

**Authors:** Ricardo Santa, Thomas Tegethoff, Alvaro Moncada, Diego Morante

**Affiliations:** 1 Colegio de Estudios Superiores de Administración-Cesa, Bogotá, Colombia; 2 Escuela militar de aviación - Emavi, Cali, Colombia; Universidade Federal do Tocantins, BRAZIL

## Abstract

This study investigates the specific impacts of Industry 4.0 technologies—such as artificial intelligence, Internet of Things (IoT), and data-driven automation—on collaboration, communication, service efficiency, and organizational performance within high-complexity hospitals in Colombia. Despite growing global interest, empirical evidence from developing countries remains scarce. To address this gap, we conducted a survey involving 272 respondents—comprising administrative personnel, healthcare providers, and supply chain staff—across hospitals classified as Level III and IV in complexity under Colombian healthcare regulations. The data were analyzed using Structural Equation Modeling (SEM) to examine hypothesized relationships between technological innovation and operational variables. Results demonstrate that Industry 4.0 technologies significantly enhance internal collaboration and communication among stakeholders, contributing to improved organizational performance. However, the anticipated reduction in patient service time through technology adoption was not statistically supported, highlighting challenges in the integration and usability of these technologies. Barriers such as inadequate staff training, limited interoperability, and financial constraints were identified as critical obstacles. The model confirms the role of collaboration in reducing service times and underscores the need for targeted strategies to fully leverage technological investments. By articulating clear relationships and practical implications, this study offers evidence-based guidance for healthcare managers, policymakers, and technologists seeking to optimize the deployment of Industry 4.0 tools in resource-constrained healthcare environments..

## Introduction

Technological innovation initiatives are pivotal in enhancing processes and products, thus significantly impacting organizational performance and effectiveness [1]. Given this, the healthcare sector must remain attuned to evolving technological landscapes, particularly across all facets of patient care [2].

The integration of Industry 4.0 technologies—a term encompassing advanced digital tools such as the Internet of Things (IoT), artificial intelligence (AI), big data analytics, machine learning, cloud computing, and robotics—has emerged as a transformative force across sectors. In the context of healthcare delivery, these technologies promise to enhance the efficiency, accuracy, and responsiveness of medical services by enabling real-time data collection, predictive diagnostics, personalized treatment plans, and streamlined workflows. Specifically, this study focuses on their implementation in high-complexity healthcare institutions, defined in Colombian regulation (e.g., Law 100/93 and Resolution 5261/1994) as Level III and IV hospitals that deliver specialized and often multidisciplinary care involving surgical interventions, intensive care units, and complex diagnostics requiring specialist oversight [3].

The healthcare system in Colombia, although ranking favorably in service accessibility and coverage (e.g., 16th globally in the 2019 WEF Competitiveness Report), continues to face pressing challenges. These include inefficiencies in communication among staff and between providers and patients, long service times, limited interoperability of digital systems, and underdeveloped innovation capabilities (ranking 77th globally in innovation capacity according to the same report). Moreover, recent global disruptions such as the COVID-19 pandemic have underscored the urgent need for resilient and adaptive healthcare infrastructures supported by advanced technologies. Also, the Global Innovation Index 2023 from the World Intellectual Property Organization (WIPO) shows Colombia as 66th among 132 countries.

While substantial research exists on Industry 4.0’s application in manufacturing and logistics, its integration within healthcare systems in developing countries remains underexplored. Few studies provide empirical validation of how these technologies influence key operational dimensions such as internal collaboration, patient communication, service delivery time, and overall institutional performance. Even fewer consider contextual barriers specific to low- and middle-income countries, such as technological adoption lag, staff training limitations, and systemic underinvestment.

This study aims to address this gap by examining the relationships between the adoption of Industry 4.0 technologies and three critical operational variables—collaboration among healthcare staff, communication with patients, and patient service time—as well as their collective impact on organizational performance in Colombian high-complexity hospitals. Examples of relevant technologies include telemedicine platforms that allow real-time consultations, AI-driven diagnostic systems capable of identifying anomalies in imaging with greater accuracy, and integrated Electronic Health Record (EHR) systems that facilitate faster information access across departments.

Therefore, it is necessary to evaluate the impact of technological innovation in the Colombian healthcare sector, and this study aims to answer the following question: What is the impact of technological innovation on variables that are part of the performance of the health sector in Colombia, such as communication, patient service time and collaboration?

Accordingly, the research objectives are: (1) to assess the impact of Industry 4.0 technologies on collaboration, communication, and service time; (2) to determine the extent to which these variables mediate improvements in organizational performance; and (3) to identify potential barriers in the integration of such technologies in high-complexity healthcare environments in Colombia.

This inquiry is of high practical relevance for healthcare administrators, policymakers, and technology providers, offering actionable insights into how digital transformation can be harnessed to improve health outcomes, resource efficiency, and service responsiveness. For instance, improving collaboration between administrative and clinical staff using shared digital dashboards can reduce redundancies in patient management, while automated scheduling systems can shorten waiting times, enhancing patient satisfaction.

To explore these dynamics, we employed a quantitative methodology based on a survey administered to 272 healthcare professionals across Level III and IV hospitals in Colombia’s southwestern region. Structural Equation Modeling (SEM) was used to test a hypothesized model linking technological innovation with the operational and performance-related constructs. The results offer a rigorous, data-driven evaluation of the real-world effects and limitations of Industry 4.0 adoption in the Colombian healthcare context.

### Literature review

The integration of Industry 4.0 technologies in the healthcare sector can be theoretically understood through several interrelated frameworks that explain how organizations adopt and benefit from technological innovation. This study draws on the Technology Acceptance Model (TAM) [4], Diffusion of Innovations Theory (DoI) [5], Resource-Based View (RBV) [6], and Organizational Change Theory [7], which together provide a robust conceptual lens for examining the mechanisms through which technological tools influence operational and organizational outcomes.

### Technological innovation and industry 4.0

Technological innovation is a significant factor in the development of any organization. Innovation improves competitiveness in the supply of products and services and generates efficiency in production processes, contributing to sustainability [8–10]. Advances in innovation have allowed humanity to reach the so-called fourth Industrial Revolution. The first industrial revolution was characterized by the beginning of large-scale production through the steam engine. The Second Industrial Revolution increased productivity through new energy sources such as electricity and oil, thus allowing the replacement of steam engines. The third Industrial Revolution began with the digitalization of production and information technologies.

The fourth industrial revolution concept is based on a German government program to maintain the manufacturing sector’s competitiveness. *Industry 4.0* is fundamental to integrating the entire production chain or value chain through information technologies, minimizing human intervention. Therefore, the production processes of products or services would become more efficient and faster. Although different authors refer to this industrial revolution as a 3.5 revolution and not as the 4th, the concept has crossed the limits of the manufacturing sector, moving to other economic sectors, such as health, construction, or even music 4.0. The benefits of implementing Industry 4.0 concepts include flexibility in production, speed, optimized logistics, resource conservation, and utilization of all available data [11–13].

Previous studies have demonstrated the positive impact of Industry 4.0 technologies on healthcare outcomes, particularly the health sector benefits from implementing Industry 4.0 initiatives. High-end examinations with augmented reality, horizontal and vertical integration of the respective data to a patient’s consultation or operation, and robotics in diagnostic management or invasive surgeries are just some benefits for the health service provider and the patient [11,14,15]. The integration of information about a patient and its immediate availability allows for the reduction of possible adverse events, which, in the worst case, can end with the death of the patient [2,16].

The Technology Acceptance Model [4] posits that perceived usefulness and perceived ease of use drive the acceptance and use of new technologies. This framework is particularly relevant in healthcare settings, where the integration of digital tools such as IoT devices, AI-powered diagnostic tools, or telemedicine platforms requires user acceptance by both administrative and clinical staff. Complementing TAM, Rogers’ Diffusion of Innovations Theory [17]explains how new technologies spread through a social system over time, influenced by innovation characteristics (e.g., relative advantage, compatibility), communication channels, and organizational culture. In the Colombian healthcare context, slow diffusion and adoption challenges are common due to infrastructural constraints and limited training, making these theories particularly pertinent.

However, the impact of the industry 4.0 concept is not only relevant to the patient. Care staff, doctors, and nurses also benefit from the possibility of immediate and accurate information access, minimizing the chances of making an error in the patient’s treatment. Also, personalized treatment according to the patient’s needs is achievable and thus reduces the patient’s time in the hospital and therefore reduces costs for both the user and the hospital [18–20].

### Collaboration

Collaboration is an exchange exercise that benefits all participating actors. Personnel collaborates to receive and share knowledge, resources, or technologies from outside the organization. Through collaboration, companies can acquire skills, knowledge, and resources efficiently and faster, as developing them internally in the organization is a long process and requires high investments. Collaboration becomes even more relevant in markets characterized by accelerated technological changes, such as the software industry. [21]. Collaborative interactions can occur through face-to-face conversations, meetings, or technological devices [22,23].

Collaboration in healthcare involves cooperative relationships among diverse stakeholders, clinical staff, administrators, and supply chain managers—who work together to achieve shared goals. This study conceptualizes collaboration through the lens of Social Exchange Theory (SET), which posits that interpersonal and interorganizational relationships are governed by reciprocal exchange of resources, trust, and mutual benefit [24]. In high-complexity hospitals, collaboration enables the sharing of knowledge and coordinated patient care, which is vital when integrating disruptive technologies like AI or EHR systems. Moreover, Resource Dependence Theory (RDT) [25] complements this view by arguing that organizations engage in collaborative relationships to access critical external resources—such as expertise or technology—that they cannot efficiently produce internally. In the Colombian healthcare context, where institutions often face funding and infrastructure gaps, collaboration becomes a strategic necessity to support Industry 4.0 adoption and optimize care outcomes.

There exist two types of Collaboration: One focuses on internal integration, which refers to the degree to which an organization defines and manages its strategies, actions, and collaborative processes to meet the needs of its customers, and second, a collaboration that focuses on external integration, which refers to the degree to which companies cooperate with their partners to shape joint strategies, practices, and processes [26,27]. Knowledge, as a primary source for innovation, should be identified according to its origin and managed since it can come from internal sources of the organization, such as employees, or external sources, such as government institutions, academics, consultants, and research institutes, and why not from civil society in general [28–30].

Internal integrations, reduction or elimination of barriers between functions, and promotion of cooperation are some of the benefits of collaboration; meanwhile, the benefits of external collaboration include improvement in process time, in the development cycle, responsiveness, and product development processes. It should be noted that innovation is increasingly a networked process, so the environment, whether local or not, is crucial to the result [31–33].

Both internal and external collaboration activities are recognized within the health services environment, such as solving scientific problems and integrating experts in different areas of health to solve a new disease or disease with unknown symptoms.

Collaborative networks are defined as links between organizations. The main aim is to acquire and integrate the skills and knowledge necessary to develop complex technologies and launch them into the market [34]. They are also long-term relationships between partners who cooperate in an environment of trust [34,35]. Collaborative networks are also recognized as interrelationships that make exchanging information, knowledge, and resources possible through mutual participant learning. Regarding their operation and structure, networks are characterized by the hierarchy levels among their members. Vertical networks are standard in customer-supplier relationships, and horizontal relationships between participants are associated with actors at the same market level [35,36].

The creation of networks is mainly due to strategic interests to find advantages from sharing technologies and knowledge and not primarily by aspects associated with costs [37]. Therefore, the network participants aim to pool resources such as knowledge or capital, sharing the risk of new development projects [21]. One of the main advantages of creating networks is acquiring complementary resources. Sternberg [38] states that the external effects of networking are remarkably incremental if the type of relationship is horizontal, less hierarchical, and trustworthy. Additionally, the literature states that interacting with external agents (even if this interaction does not lead to the establishment of cooperation agreements) has a substantial impact on the increase of innovation initiatives, as it helps to improve the knowledge, skills, and experience of employees [31,33].

Consequently, developing external relations increases access to resources and improves the response to market requirements. [39]. Finally, different authors agree that inter-organizational networks provide access to a broader range of information and resources that individual companies could not achieve, resulting in higher performance [21,40].

The literature identifies six benefits companies can receive from network participation concerning increasing innovation indicators. These were shared risk, access to new markets and technologies, increased speed of commercialization, accumulation of complementary knowledge, protection of property rights, and the development of skills increasing the use of external knowledge [41]. For a better understanding of the effect of collaborative networks on innovation, not only their density should be analyzed (in terms of the number of agreements or the different types of agents with which the Company cooperates) but also the depth of such networks (in terms of the types of agreements employed: short-term versus long-term, or a combination of both), and its operational performance (i.e., the operation of the network in terms of planning, working methods, engagement, monitoring, and evaluation). [42]

Consequently, we propose the following hypotheses:

**H1:** *There is a positive impact between Industry 4.0 and collaboration among stakeholders*

### Communication with users

Effective communication—defined here as the clarity, accuracy, and timeliness of information exchange between healthcare personnel and with patients—is central to organizational coordination and service quality. This variable is first guided by the Shannon-Weaver Model [43], a foundational theory in communication studies that views communication as a process involving an information source, a transmitter, a channel, and a receiver, with potential for noise and distortion. This model is highly relevant in healthcare systems where errors in message transmission (e.g., miscommunication about patient status) can lead to adverse outcomes. Additionally, Media Richness Theory [44] helps explain the appropriateness of different communication media (e.g., face-to-face, phone, digital platforms) depending on message complexity. In hospitals transitioning to Industry 4.0 technologies, digital tools (e.g., telemedicine, automated notifications) vary in their media richness and must be aligned with clinical communication needs to avoid breakdowns in patient care.

Technological advances and innovation have been the main drivers in developing organizations’ capacity to transmit internal and external information [45–47]. Technologies that involve patient communication and processes may save costs, but their impact on health outcomes is largely unknown. The rapid advancement of technology has impacted the health sector much faster and cheaper than other sectors, such as manufacturing [48–50]. Innovative technologies, such as databases, systematization of medical records, diagnosis algorithms, and telemedicine systems, are used to monitor patient health and minimize safety risks [51].

International organizations such as the World Health Organization (WHO) have called for adopting new technological tools in the healthcare sector to benefit patients and stakeholders. Especially when there are spaces and distances, the use of information and communication technologies for information exchange is vital in streamlining the processes that involve diagnosis, treatment, health promotion, and disease prevention [52].

Given these arguments, we propose the following hypotheses

**H2:** *There is a positive impact between Industry 4.0 and Communications*

### Customer service time

Currently, healthcare services are complex and expensive to operate. Efficient service time is a crucial metric for evaluating the effectiveness of healthcare delivery and patient satisfaction. However, innovative technologies that improve information management and quality can reduce operations complexity and costs. A significant advantage of using technology in the healthcare sector is that it can boost the interoperability of healthcare databases, providing greater access to patient medical records, device tracking, prescription databases, and hospital assets, reflected in time and cost-benefit [53,54].

Service time refers to the duration a patient spends in receiving medical services, from initial contact to resolution. In this study, it is analyzed using the principles of Lean Management and Six Sigma, both of which aim to reduce waste, eliminate inefficiencies, and optimize service delivery in operational workflows [55,56]. The relevance of these approaches in healthcare has been widely acknowledged, particularly in settings like emergency departments or surgical units where time is a critical resource. Moreover, Queuing Theory [57] provides a mathematical foundation for analyzing bottlenecks and wait times in service systems. Together, these theories support the investigation of how Industry 4.0 tools—such as real-time tracking systems, AI-assisted triage, or scheduling algorithms—can be leveraged to reduce delays, streamline patient flow, and enhance the overall efficiency of complex healthcare environments.

Today, the Internet and other technological facilities allow for the reduction of services, lowering cost and increasing quality, boosting efficient supervision of management, and monitoring events of interest in public health. Consequently, more attention has been paid to integrating all these technologies into medical care, given the demands on time and emergency diagnoses such as those experienced in the current SARS-CoV-2 pandemic [58]. However, it remains a challenge to understand the problems and opportunities arising from health care in terms of care time and recovery. Taking into account that the costs of medical care are increasing and time is a significant factor, it is essential to include in health services technological developments that allow for improvement and managing economic and time expenses [59–61]. We propose, therefore, the following hypothesis for this study:

**H3:** *There is a positive impact between Collaboration and Customer Service Time.*

**H4:** *There is a positive impact of Communication on Customer Service Time*

**H5:** *There is a positive impact of Industry 4.0 on Customer Service Time*

### Organizational performance

According to Gopalakrishnan [62], organizational performance can be defined based on different factors. These include, among others, a) efficiency, which is related to resource inputs and outputs; b) effectiveness, which is related to business growth and employee satisfaction; and lastly, c) financial results, which are related to return on assets, investment, and growth. Other authors claim that organizational performance depends on the objective of a company and can be measured in terms of asset profitability and operating profit. Growth associated with sales, market segment, or new product development; and customer and employee satisfaction, which is related to morale and wellbeing, sales, profitability, number of new products, new product sales segment, market segment, return on capital, rate of return, operating profit, profit margin [63–65]. Meanwhile, Koo et al. [66] measure organizational performance based on six attributes: operating revenue, profit margin, growth in the number of employees, return on assets, return on equity, and sales growth. Lastly, it is worth highlighting Nakata’s [67] definition of this construct, bringing together a set of financial indicators (sales and financial and operational profit), issues relating to quality and customer satisfaction (retention rate), and innovation-related indicators (success of new products or services). According to Lee and Choi [68], organizational performance is how companies accomplish their business objectives, measured in continuous learning, profit, or other financial benefits. Organizational performance can also be interpreted through the Balanced Scorecard Framework [69], which expands traditional performance metrics to include financial, customer, internal process, and learning & growth perspectives. This holistic approach is particularly relevant in healthcare organizations, where outcomes depend not only on cost-effectiveness but also on service quality and workforce engagement.

Additionally, Contingency Theory [70] argues that organizational effectiveness depends on the alignment between internal capabilities and external conditions. In the context of Industry 4.0 implementation, this means that the success of technology integration depends on organizational structure, staff readiness, and environmental complexity. Additionally, General Systems Theory [71] provides a meta-theoretical perspective, framing healthcare organizations as open systems where technological, human, and procedural elements interact dynamically. This systems perspective underpins the study’s integrative model, linking Industry 4.0 with communication, collaboration, and service time to explain performance.

Sustainable competitive advantage is the consequence of superior organizational performance, which results from intangible assets such as knowledge learned and assimilated. The stock of internally developed resources is the result of a combination of the current resources and capabilities of an organization for creating new resources that generate a market offering of an increased value, a higher performance, and a more significant competitive advantage that is sustainable over time [72,73].

Performance remains the fundamental objective for organizations striving to endure in a dynamic market environment. Enhancing performance necessitates the adoption of suitable strategies along with the effective management of technological and human resources. Operational performance refers to the degree to which firms enhance outcomes in areas such as cost, quality, delivery, and flexibility [74]. Attaining operational performance reflects a company’s competitive capabilities, enabling it to achieve superior competitive strength compared to its primary competitors within target markets. In the context of sustainability, operational performance becomes increasingly vital, serving as a baseline requirement in a competitive marketplace [75].

**H6:** *There is a positive impact on Communication between stakeholders and Organizational Performance*

**H7:** *There is a positive impact of Industry 4.0 on Organizational Performance*

**H8:** *There is a positive impact of user Service Time on Organizational Performance*

**H9:** *There is a positive impact of Collaboration on Stakeholders and Organizational Performance.*

**Fig 1:**
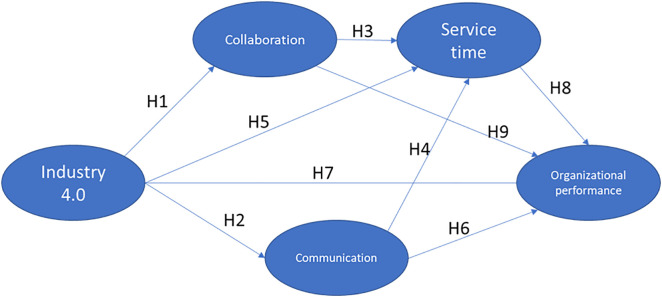
Hypothetical model.

Fig 1 shows the hypothetical model:

## Methodology

This study aims to evaluate prespecified collaborative relationships, communication, and service time in performance and their relationship to technological innovations. This research is confirmatory in explaining and quantifying the relationships between variables and determining the causes [76,77]. There is no evidence of the factors that influence performance in the health sector in Colombia after the implementation of technological innovations.

To test the hypotheses shown in Figure No. 1, all research and measurement instruments, such as the survey instrument, measurement constructs, and best-fit model, were developed, considering the parameters given by Hair et al. [78]. A questionnaire was designed based on an extensive literature review to collect information from administrative and care personnel operating high-impact hospitals in the southwest region of Colombia.

The denomination used for the hospitals surveyed as having a high level of complexity has been used since before Law 100/93, with an initial antecedent in Law 10 of 1990 and Decree 1760 of 1990. These definitions are adjusted after 1993 and Resolution 5261 of 1994, where the precision on the Levels of Complexity is made, establishing the following:

- **Level I: General practitioner and/or auxiliary and/or** paramedical personnel and/or other non-specialized health professionals.
- **Level II**: General practitioner and/or paramedical professional with inter-consultation, referral, and/or advice from specialized personnel or resources.
- **Level III and IV**: Medical specialist with the participation of the general practitioner and/or paramedical professional.

Within these levels of complexity, the levels of surgical care are established, taking responsibility for the different levels of complexity and different levels of care.

The survey format consisted of a demographic part, and the set of variables of interest that allow the construction of the model described in Figure No.1. A five-point Likert scale (Strongly agree – Strongly disagree) was used to rate statements related to the operationalization of model variables. Taking into account the advantages of online surveys [79], an electronic survey was developed, and link access was shared electronically. Survey was conducted between July 2023 and July 2024

To examine potential common-method bias (CMB), we conducted Harman’s single-factor test, following the recommendations of Bozionelos and Simmering [80]. An exploratory factor analysis including all measurement items was performed using the unrotated principal component method. The results showed that the first factor accounted for 32% of the total variance, which is well below the 50% threshold, indicating that CMB is not a critical concern in this study.

To examine potential non-response bias, we compared early respondents (first 30% of responses) with late respondents (last 30%) following the approach suggested by Armstrong and Overton [81]. Independent-sample t-tests were conducted on the main constructs (Industry 4.0 technologies, collaboration, communication, service time, and organizational performance). The results showed no statistically significant differences between early and late respondents (p > 0.05 for all constructs), indicating that non-response bias is unlikely to affect the study’s results.

Prior to beginning the survey, participants were informed of their right to withdraw at any point without penalty. Informed consent was obtained from all respondents, with consent being verbal and implied by their decision to proceed with the online survey after receiving the detailed information sheet. The study was conducted with adult participants; therefore, parental or guardian consent was not applicable. The study protocol received ethical approval from the Ethics Committee of the Colegio de Estudios Superiores de Administración (CESA). The committee did not exempt the study from the requirement of informed consent. We reviewed the integrity of each questionnaire and excluded 57 from the present analysis due to inconsistencies and significant missing data. Valid data (272 surveys) were analyzed using confirmatory factor analysis.

**Fig 2:**
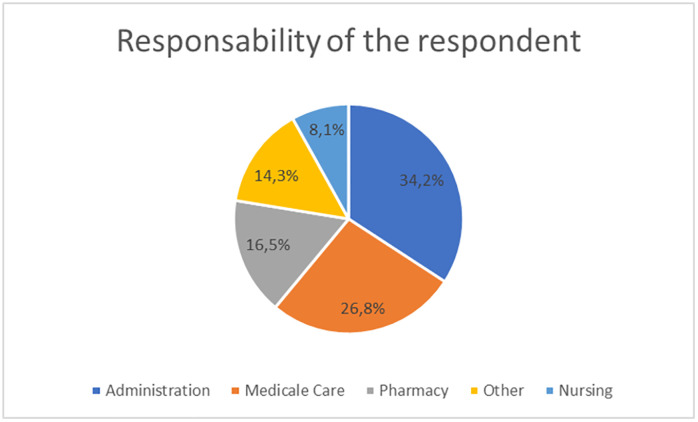
Area of Responsibility of the respondent.

Figure No. 2 shows the respondent’s areas of responsibility. For this study, 93 respondents are related to administrative positions, equivalent to 34.2%, 73 people are associated with healthcare areas 26.8% of respondents, and 16.5%, equal to 45 people, work in the pharmacy supply chain (Fig 2).

SPSS and AMOS software were used for the analysis and multivariate data. The predictive relationship of the model variables and the fit indices of the model were estimated to determine the confidence level. Confirmatory factor analysis (FCA) was used to study the relationships between observed and continuous latent variables and to determine the overall fit of the measurement model [82,83]. Factorial loads, elements loaded in a single construction (i.e., no cross-loading), and latent constructions correlated (equivalent to oblique rotation in exploratory factor analysis) were estimated. Internal consistency was examined using Cronbach’s alpha coefficient and the correlation of each element in total. Table 1 summaries the values of the coefficient of the constructions.

**Table 1:** Cronbach’s Alpha.

	Items	Alpha
Technological innovation	3	.902
Collaboration	6	.955
Communication with users	5	.910
Service time received by the user	4	.890
Organizational performance	3	.937

Source: By the authors.

The Cronbach’s alpha values presented in Table 1 indicate excellent internal consistency for all constructs, as all values exceed the commonly accepted threshold of 0.70 [84,85]. In particular, the “Collaboration” construct exhibits a very high alpha (0.955), which, while demonstrating strong reliability, may also suggest potential redundancy among its six items. This high value is partly due to the number of items, as constructs with more indicators tend to produce higher alpha values (Tavakol & Dennick, 2011). After careful review, all items were retained to ensure comprehensive coverage and content validity of the constructs.

Although six items were initially used to measure the “Collaboration” construct (as shown in Table 1), the final SEM model includes only four items. This reduction was the result of the confirmatory factor analysis (CFA), where two items were removed due to high cross-loadings, or poor contribution to construct validity. The removal of these items was necessary to improve the overall model fit and ensure that the construct measurement was both parsimonious and theoretically sound [78].

This model shows 171 moments, with 48 different parameters to estimate. Chi-square equals 350.903 with 121 degrees of freedom, with a CMIN/DF of 2.900 and a probability level 0.000. The literature suggests a ratio of approximately five or less as a reasonable criterion Wheaton et al. [86,87] for the model to be accepted. It is recommended to use ratios as low as two or as high as five and to make ratios in the range of 2:1 or 3:1 as indicators of an acceptable fit between the hypothetical model and the sample data [85] The GFI value above 0.9 is compatible with the model, with a result of 0.935 [88] In addition, the reliability of each of the constructs in the model was evaluated using several fit statistics and the mean square approximation error (RMSEA), which was acceptable as the model had a value of 0.082 and less than 1.0 [88,89] The reference comparison of the adjustment indices suggests that the hypothetical model fits well with the variance-covariance matrix observed concerning to the null or independence model (see Table 2).

**Table 2:** Baseline comparison.

Model	NFI	RFI	IFI	TLI	CFI
Default template	.928	.909	.952	.939	.952
Saturated model	1.000		1.000		1.000
Standalone model	.000	.000	.000	.000	.000

Source: By the authors.

## Results

The findings of this study underscore the significant impact of technological innovations on various aspects of organizational performance and user interactions in the healthcare sector. The Structural Equation Modeling (SEM) results reveal insights that align with and sometimes contradict existing literature.

First, the strong positive relationship between technological innovations (Ind 4.0) and user communication (b = 0.59, p < 0.001) confirms Hypothesis H2. This result is consistent with prior studies emphasizing that technological advancements are crucial for enhancing user communication channels and fostering better customer relations and engagement. Integrating innovative technologies such as e-business platforms and Internet-based solutions facilitates more effective and efficient communication with users.

However, the study found a low and statistically insignificant relationship between technological innovations and service time (b = −0.25, p = 0.095), leading to the rejection of Hypothesis H5. This outcome suggests that contrary to the expected efficiency gains from technological advancements, the current implementation of these technologies in e-business and medical solutions has not effectively reduced service times. This finding highlights a potential gap in the practical application of these innovations, possibly due to inadequate integration or training, which may render them inefficient and uncomfortable for users. This fact contrasts with the theoretical benefits proposed in existing literature [90,91].

Hypothesis H7 was supported by the significant positive relationship between technological innovations and organizational performance (b = 0.26, p < 0.001). This result aligns with the broader consensus in the literature that technological advancements enhance organizational efficiency and overall performance [92]. Similarly, the confirmation of Hypothesis H1 (b = 0.56, p < 0.001) emphasizes the pivotal role of technological innovations in fostering collaboration among employees, which is crucial for achieving high-impact organizational.

The significant relationship between collaboration and service time (b = 0.63, p < 0.001), as well as between collaboration and organizational performance (b = 0.46, p < 0.001), supports Hypotheses H3 and H9, respectively. These findings corroborate the importance of collaborative practices in reducing service times and enhancing overall performance in healthcare organizations. Effective collaboration, facilitated by technology, enables more streamlined operations and better resource management [93].

Conversely, Hypothesis H6, which proposed that communication with users predicts organizational performance, was not supported (b = 0.08, p = 0.137). That finding suggests that, despite the advancements in communication technologies, there may be a lack of adequate training or integration of these technologies to impact organizational performance significantly. Consequently, more focused training and technology integration efforts are needed to realize the full potential of user communication enhancements.

**Fig 3:**
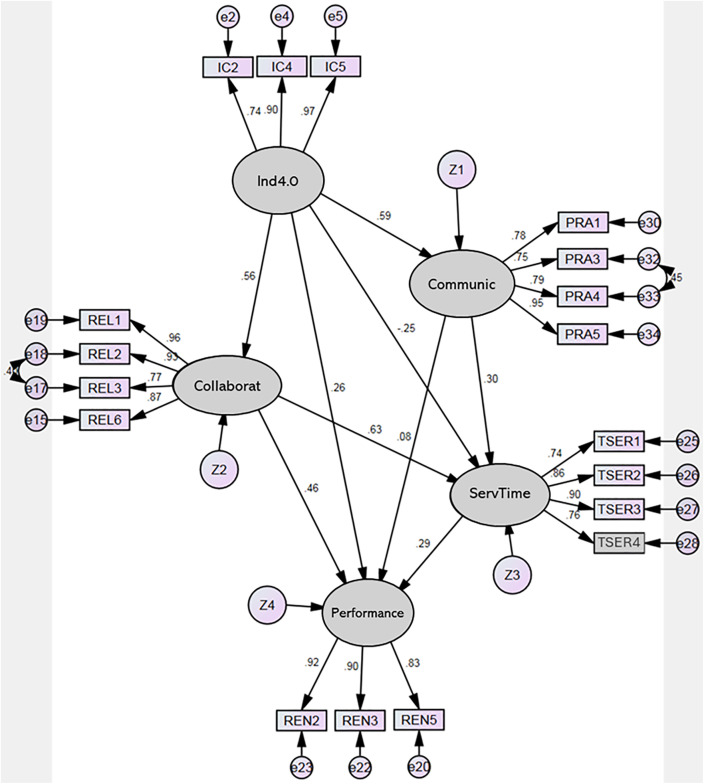
Structural Model.

Lastly, the confirmation of Hypothesis H8 (b = 0.29, p < 0.001) highlights the critical role of service time in organizational performance. Efficient service delivery is crucial to operational success and customer satisfaction. Additionally, the significant relationship between communication with users and service time (b = 0.30, p < 0.001), supporting Hypothesis H8, underscores the necessity of effective communication management to improve service delivery times and, consequently, organizational performance (Table 3) (Fig 3).

**Table 3:** Regression Weights:(Group number 1 – Default model).

			Estimate	SE.	CR.	P	Label
Communic	<---	Ind4.0	.471	.056	8.447	***	H2: Confirmed
Collaborat	<---	Ind4.0	.487	.056	8.714	***	H1: Confirmed
ServTime	<---	Ind4.0	−.151	.052	−1.830	.097	H2: Not confirmed
ServTime	<---	Communic	.217	.055	3.933	***	H4: Confirmed
ServTime	<---	Collaborat	.429	.054	7.933	***	H3: Confirmed
Performance	<---	Ind4.0	.215	.054	3.999	***	H7: Confirmed
Performance	<---	Communic	.082	.055	1.488	.137	H6: Not confirmed
Performance	<---	Collaborat	.437	.061	7.199	***	H9: Confirmed
Performance	<---	ServTime	.412	.077	5.323	***	H8: Confirmed

**Note:** *** significance < 0.001.

Source: By the authors.

### Participant consent

In this study, informed consent was obtained from all participants. The consent was written and included as part of the survey form. No minors were involved in the study; therefore, parental or guardian consent was not required. The entire procedure, including the consent process, was reviewed and approved by the Ethics Committee of CESA.

## Discussion

The findings of this study confirm that Industry 4.0 technologies significantly influence collaboration, communication, and organizational performance in Colombian high-complexity hospitals, while the direct impact on service time remains limited. These results are consistent with the perspectives of Bag et al. [15], who emphasize the critical role of digital tools such as big

data analytics and automation in improving healthcare coordination and efficiency. Dubovitskaya et al. [53] also supports the idea that integrated digital platforms enhance communication and collaboration among hospital teams, aligning with the results obtained in this research.

In contrast to the positive effects identified on collaboration and communication, the anticipated reduction in service time was not fully supported. This discrepancy could stem from operational barriers such as insufficient training or incomplete integration of digital workflows, as suggested by Avila-Tomás et al. [11] and Kim and Han [18]. Despite this, our results indicate that collaboration and communication have indirect effects on service time, demonstrating that organizational dynamics act as mediators between technology adoption and process improvements.

The strong influence of collaboration on organizational performance echoes the findings of Cruz et al. [22] and Schilling [21], who highlight teamwork and coordinated processes as key drivers of success. Moreover, this study aligns with the observations of Bharadwaj et al. [90] and Santa et al. [74], who note that digital transformation enhances strategic outcomes when paired with organizational readiness and knowledge sharing. These findings reinforce the argument that Industry 4.0 adoption must be combined with cultural and structural changes to realize its full benefits.

Technological advancements, particularly those associated with Industry 4.0, are crucial in enhancing communication and collaboration among healthcare personnel, including users, administrators, and care staff, thereby significantly affecting organizational performance. However, the results reveal that the technologies employed, and the medical outcomes achieved through information systems are not delivering the expected service times for users. Consequently, these technologies fail to meet their intended objectives, contradicting technological innovation’s theoretical and practical aims. This discrepancy highlights the critical need for adoption and integration strategies that enable staff to fully capitalize on these technological advancements.

Moreover, technological innovations demonstrate predictive power in enhancing collaboration among personnel and improving relationships during service delivery, affirming the pivotal role of collaboration within healthcare organizations, which is a key determinant of organizational performance.

Regarding communication, there is a pressing need to strengthen training in the use of new technologies to enhance service quality and exert a more substantial impact on organizational performance. The positive correlation between technological innovations and user communication underscores the importance of integrating advanced technologies to improve communication channels and foster better customer engagement. However, the absence of a significant relationship between technological innovations and service time suggests a potential gap in the practical application of these technologies, indicating that current implementations may not be effectively reducing service times.

In terms of the relationship between technological innovations and organizational performance, it is important to note that the positive impact of technological innovations on organizational performance is consistent with existing literature, reinforcing the notion that technology enhances organizational efficiency.

The strong association between technological innovations and collaboration underscores the critical role of technology in fostering collaborative practices, which are indispensable for achieving high-impact outcomes. Technological advancements facilitate seamless communication, resource sharing, and coordinated efforts among team members, thereby enhancing the overall efficiency and effectiveness of collaborative endeavors.

Empirical evidence indicates that collaboration significantly reduces service time, highlighting the importance of cooperative efforts in streamlining operations. By fostering a collaborative environment, organizations can optimize workflow processes, minimize delays, and improve the timeliness of service delivery, ultimately enhancing customer satisfaction and organizational efficiency.

Moreover, the significant positive correlation between collaboration and organizational performance underscores the fundamental importance of teamwork in achieving organizational goals. Collaborative efforts enable the pooling of diverse skills, knowledge, and perspectives, thereby driving innovation, improving decision-making, and enhancing the organization’s ability to achieve its strategic objectives.

However, the observed lack of a significant relationship between communication with users and organizational performance suggests there is a pressing need for better integration and more comprehensive training in the use of communication technologies. Without effective integration and user training, the potential benefits of advanced communication tools may remain untapped, limiting their impact on organizational outcomes.

The confirmation that service time has a direct impact on organizational performance further emphasizes the critical importance of efficient service delivery. Timely and efficient service is a key determinant of customer satisfaction and, consequently, overall organizational success. Thus, organizations must prioritize strategies that reduce service time to enhance their performance.

Lastly, the positive relationship between communication with users and service time highlights the essential role of effective communication management in improving service delivery times. Clear, timely, and efficient communication with users not only enhances their experience but also contributes to the swift resolution of issues, thereby reducing overall service time and enhancing organizational performance.

## Conclusion

This research demonstrates that the implementation of Industry 4.0 technologies improves collaboration and communication, which in turn enhance hospital performance. However, the lack of a significant direct effect on service time suggests that the benefits of these technologies depend on complementary factors such as staff training, workflow redesign, and cultural adaptation.

The study contributes to the literature by extending Industry 4.0 research from Bag et al. [15] to the healthcare sector in developing countries, providing empirical evidence of its impact on organizational performance. From a managerial perspective, the results highlight the importance of aligning digital strategies with collaborative practices and staff engagement to achieve operational benefits.

Future research should examine longitudinal impacts of Industry 4.0 adoption and investigate the challenges of integrating these technologies into existing hospital workflows, including the resistance to change and infrastructural gaps identified by Avila-Tomás et al. [11] and Kim and Han [18].

### Practical implications

For Managers: For managers, effective integration of technological innovations and comprehensive training are essential to achieve intended efficiencies. This includes adopting new technologies and ensuring staff proficiency through ongoing training programs. Emphasizing collaborative practices enhances service delivery and organizational performance by fostering coordinated care and reducing errors. Managers should promote a culture of collaboration through regular interdepartmental meetings and knowledge-sharing sessions. Additionally, improving communication strategies and tools, such as telemedicine and secure messaging systems, is crucial for enhancing service efficiency. By focusing on technology integration, training, collaboration, and communication, managers can create a more efficient, collaborative, and effective healthcare environment, ultimately leading to better patient care and satisfaction.

For Patients: Technological innovations can significantly enhance the patient’s experience by providing faster, more accurate care and facilitating better communication with healthcare providers. These technologies can lead to more personalized treatment plans and higher overall patient satisfaction. Evaluating how technological innovations affect accessibility and quality of service is essential to ensure that all patients, regardless of their location or socioeconomic status, receive optimal care. Implementing user-friendly technologies can make healthcare services more accessible and efficient, ultimately improving patient outcomes. Incorporating robust patient feedback mechanisms can help healthcare providers understand the effectiveness of new technologies from the patient’s perspective, enabling continuous improvement and ensuring that the technologies meet patient needs and expectations

For academics: For academics, it is crucial to explore the barriers to effective technology integration in healthcare and develop frameworks to overcome these challenges. Understanding how technological innovations interact with organizational dynamics can provide a holistic view of their impact. Additionally, creating educational programs that cover both the technical and managerial aspects of these innovations is essential. By addressing these areas, academics can significantly contribute to the successful adoption and implementation of new technologies, ultimately improving healthcare delivery and patient outcomes.

For governmental institutions: Governments should formulate policies that facilitate the effective implementation of technological innovations, especially in critical sectors such as healthcare. Providing incentives for organizations to invest in training programs is vital for enhancing the practical application of new technologies. Furthermore, establishing regulations and standards is essential to ensure the efficient and ethical use of technological advancements across various industries. By focusing on these areas, governments can create an environment that promotes innovation while safeguarding public interests.

In essence, while technological innovations and collaboration significantly enhance communication, service time, and overall performance, there is a need for improved integration and training to maximize their benefits. Effective integration ensures that these technologies are seamlessly embedded into existing workflows, reducing disruptions and enhancing efficiency. Comprehensive training programs are crucial to equip healthcare personnel with the necessary skills and knowledge to utilize these technologies fully.

Future research should explore the barriers to effective technology implementation and develop strategies to address these challenges. Future research should delve into identifying and understanding the barriers to effective technology implementation in healthcare settings. These barriers could include financial constraints, resistance to change among staff, lack of technical infrastructure, and insufficient managerial support. By thoroughly investigating these obstacles, researchers can provide valuable insights into the specific challenges faced by healthcare organizations.

### Limitations

This study faces several limitations that could affect the interpretation and generalizability of its findings. First, the reliance on convenience sampling may introduce bias, as the participants may not be fully representative of the broader healthcare population. Second, the study’s limited geographic scope further restricts the applicability of the results to other regions or healthcare systems, potentially limiting the broader relevance of the conclusions drawn. Additionally, the use of self-reported data introduces the risk of recall and social desirability biases, which may skew the results. Lastly, the focus on specific Industry 4.0 technologies within the healthcare sector highlights a challenge of technological variability; as these technologies rapidly evolve, the findings may quickly become outdated or may not apply to emerging innovations in the field.

## Appendix A

### The survey instrument and source

This is a list of the questions used for each construct and its academic support.

### Industry 4.0 Technologies

1. Our hospital has implemented advanced digital technologies (e.g., Internet of Medical Things (IoMT), artificial intelligence, or robotics) to support patient care and administrative processes
2. Real-time data analytics and monitoring systems are extensively used in our hospital to improve patient treatment and operational decision-making.
3. Our hospital’s clinical and administrative systems (e.g., electronic health records, telemedicine platforms) are interconnected and integrated.
4. We use intelligent automation (e.g., AI-assisted diagnostics, robotic surgery, automated scheduling) to enhance efficiency and accuracy in patient care.
5. Our hospital has invested in cloud-based platforms or digital infrastructures to ensure secure and scalable access to patient and operational data.
6. The adoption of Industry 4.0 technologies has improved the flexibility and customization of patient treatments and hospital services.

### Communication

1. Communication among healthcare staff (doctors, nurses, administrative staff) in our hospital is clear and effective.
2. Important information about patients or hospital operations is shared in a timely manner across departments.
3. The hospital uses appropriate communication tools (e.g., telemedicine, secure messaging, internal platforms) to ensure accurate information flow.
4. There is open communication between healthcare staff and patients, ensuring that patients receive clear and understandable information.
5. Digital technologies (e.g., EHR systems, automated alerts) have improved the quality and reliability of communication among hospital staff.

### Collaboration

1. Healthcare staff (administrative, clinical, and supply chain teams) collaborate effectively to achieve common goals.
2. There is an active exchange of information and resources between departments to improve patient care and operational efficiency.
3. Digital platforms (e.g., electronic health records, shared dashboards) enhance collaboration between different hospital units.
4. Collaboration between hospital staff and external partners (e.g., suppliers, laboratories) is efficient and well-coordinated.
5. Collaborative decision-making among healthcare staff improves the speed and quality of patient services.

### Service Time

1. Our hospital has reduced patient waiting times due to the adoption of digital technologies (e.g., online scheduling, automated triage systems).
2. Patient admission and discharge processes are completed promptly and efficiently.
3. The use of real-time data and automated workflows has improved the speed of healthcare services.
4. Our hospital’s operational systems help minimize delays in patient care delivery.
5. Industry 4.0 technologies have improved the responsiveness of our hospital’s services.

### Organizational performance

1. The implementation of digital technologies has increased the overall efficiency of hospital operations.
2. Our hospital has experienced improved service quality and patient satisfaction due to technological innovation.
3. Operational costs have been reduced because of adopting advanced healthcare technologies.
4. The hospital has improved its adaptability and resilience in response to challenges (e.g., pandemics) through digital transformation.
5. The integration of Industry 4.0 technologies has led to a measurable improvement in hospital performance indicators (e.g., productivity, resource utilization).
